# Essential role of miR-200c in regulating self-renewal of breast cancer stem cells and their counterparts of mammary epithelium

**DOI:** 10.1186/s12885-015-1655-5

**Published:** 2015-09-23

**Authors:** Zhong-Ming Feng, Jun Qiu, Xie-Wan Chen, Rong-Xia Liao, Xing-Yun Liao, Lu-Ping Zhang, Xu Chen, Yan Li, Zheng-Tang Chen, Jian-Guo Sun

**Affiliations:** 1 Cancer Institute of PLA, Xinqiao Hospital, Third Military Medical University, Chongqing, 400037 P. R. China; 2 Department of Medical English, College of Basic Medicine, Third Military Medical University, Chongqing, 400038 P. R. China

**Keywords:** miR-200c, Breast cancer, Cancer stem cells, Carcinogenesis, Self-renewal, PDCD10, Malignant transformation

## Abstract

**Background:**

Breast cancer stem cells (BCSCs) have been reported as the origin of breast cancer and the radical cause of drug resistance, relapse and metastasis in breast cancer. BCSCs could be derived from mutated mammary epithelial stem cells (MaSCs). Therefore, comparing the molecular differences between BCSCs and MaSCs may clarify the mechanism underlying breast carcinogenesis and the targets for gene therapy. Specifically, the distinct miRNome data of BCSCs and MaSCs need to be analyzed to find out the key miRNAs and reveal their roles in regulating the stemness of BCSCs.

**Methods:**

MUC1^−^ESA^+^ cells were isolated from normal mammary epithelial cell line MCF-10A by fluorescence-activated cell sorting (FACS) and tested for stemness by clonogenic assay and multi-potential differentiation experiments. The miRNA profiles of MaSCs, BCSCs and breast cancer MCF-7 cells were compared to obtain the candidate miRNAs that may regulate breast tumorigenesis. An miRNA consecutively upregulated from MaSCs to BCSCs to MCF-7 cells, miR-200c, was chosen to determine its role in regulating the stemness of BCSCs and MaSCs in vitro and in vivo. Based on bioinformatics, the targets of miR-200c were validated by dual-luciferase report system, western blot and rescue experiments.

**Results:**

In a 2-D clonogenic assay, MUC1^−^ESA^+^ cells gave rise to multiple morphological colonies, including luminal colonies, myoepithelial colonies and mixed colonies. The clonogenic potential of MUC1^−^ESA^+^ (61.5 ± 3.87 %) was significantly higher than that of non-stem MCF-10A cells (53.5 ± 3.42 %) (*P* < 0.05). In a 3-D matrigel culture, MUC1^−^ESA^+^ cells grew into mammospheres with duct-like structures. A total of 12 miRNAs of interest were identified, 8 of which were upregulated and 4 downregulated in BCSCs compared with MaSCs. In gain- and lost-of-function assays, miR-200c was sufficient to inhibit the self-renewal of BCSCs and MaSCs in vitro and the growth of BCSCs in vivo. Furthermore, miR-200c negatively regulated programmed cell death 10 (PDCD10) in BCSCs and MaSCs. PDCD10 could rescue the tumorigenesis inhibited by miR-200c in BCSCs.

**Discussion:**

Accumulating evidence shows that there is a milignant transformation from MaSCs into BCSCs. The underlying mechanism remains unclear. In present study, miRNA profiles between MaSCs and BCSCs were obtained. Then miRNA-200c, downregulated in both MaSCs and BCSCs, were verified as anti-oncogene, and played essential role in regulating self-renewal of both kinds of stem-like cells. These findings reveal a novel insights of breast tumorigenesis.

**Conclusions:**

PDCD10 is a target gene of miR-200c and also a possible mechanism by which miR-200c plays a role in regulating the stemness of BCSCs and MaSCs.

**Electronic supplementary material:**

The online version of this article (doi:10.1186/s12885-015-1655-5) contains supplementary material, which is available to authorized users.

## Background

Accumulating evidence shows that tumors are organized in a hierarchy of heterogeneous cell populations with different biological properties and that the ability to sustain clonogenic capacity and growth exclusively resides in a small proportion of tumor cells termed cancer stem cells (CSCs) or tumor-initiating cells (TICs) [1–3]. CSCs, capable of uncontrolled growth, self-renewal and multi-lineage differentiation, are the fundamental reason for drug resistance, tumor relapse and metastasis. CSCs have been identified in blood cancer and a number of solid tumors, including breast cancer, through an experimental strategy that combines sorting of tumor cell subpopulations based on surface markers with functional transplantation into appropriate animal models [4–6]. To reduce or eliminate CSCs, it is necessary to determine the regulatory mechanism that controls the expansion and self-renewal of CSCs [7].

Scientists postulate that normal stem cells with accumulating mutations could initiate the process of carcinogenesis in a majority of tumors [8]. Normal stem cells live longer than differentiated cells and are exposed to DNA-damaging agents for a longer time, allowing for accumulation of epigenetic modifications or genetic mutations. Human embryonic stem cells (hESCs) during long-term culture acquired chromosomal changes similar to those occurring in tumorigenesis and underwent deregulation of self-renewal and dysfunction of related genes, leading to malignant transformation [9]. It is now clear that many pathways of normal stem cells, which guide cellular proliferation, differentiation and apoptosis, are also prominent in CSCs [1].

Breast cancer is one of the most common cancers in adult females, accounting for 7–10 % of solid malignant tumors, second only to cervical cancer in women. Breast cancer stem cells (BCSCs) with biomarker of ESA^+^CD44^+^CD24^-/low^ [10] have been reported as the origin of different pathological types of breast cancer and the radical cause of drug resistance, tumor relapse and metastasis in breast cancer. For the source of BCSCs, it has been proved that they could be derived from mutated mammary epithelial stem cells (MaSCs) responsible for development and damage repair of breast tissue [11–13].

The above findings support the existence of BCSCs and indicate the great significance to analyze molecular differences in gene or RNA profiles between MaSCs and BCSCs and reveal the mechanism of breast carcinogenesis. In recent years, rapid progress has been made in research on MaSCs. Shackleton et al. [14] found that a single cell within the Lin^−^CD29^hi^CD24^+^ population can reconstitute a complete mammary gland in mice. For humans, MUC1^−^ESA^+^ epithelial cells isolated from luminal epithelial cell populations of primary culture are considered as MaSCs with stem cell properties [15].

MiRNAs are endogenously expressed non-coding RNAs of 21–25 nt in length that interact with native coding mRNAs to cause mRNA translation inhibition or mRNA degradation. The regulatory roles of miRNAs in diverse developmental and physiological events, and disease pathogenesis have become evident in the last few years [16, 17]. Studies demonstrated aberrant expression of miRNAs in several human malignancies, including leukemia, lymphoma, lung cancer, hepatocellular cancer, colorectal cancer, gastric cancer and breast cancer [18, 19]. MiRNAs also play important regulatory roles in CSCs and tumorigenesis [20–25]. Therefore, CSC-specific miRNAs would provide valuable information for CSC properties, shedding new light on mechanism of diverse cancers.

To investigate the roles of miRNAs in BCSC biology, an important step is to examine miRNA profiles in BCSCs and MaSCs. We report here miRNome data of MaSCs isolated from MCF-10A, an established breast epithelial cell line. Compared with the miRNAome data of BCSCs sorted from breast cancer cell line MCF-7 in previous research [26], we discussed the key miRNAs, their essential function and possible mechanisms in regulating the stemness of BCSCs.

## Methods

### Cell culture

HeLa, NIH-3T3 and HEK-293 T cells (cryopreservation in our lab) were cultured in Dulbecco’s MEM modified medium (DMEM) with 10 % fetal bovine serum (FBS) at 37 °C and 5 % CO_2_ incubator. Before being used as feeder cells, NIH-3T3 cells were exposed to 50 Gy of ^60^Co radioactive source. Breast cancer cell line MCF-7 and normal mammary epithelial cell line MCF-10A were obtained from ATCC (Manassas, VA). MCF-7 cells were cultured in minimum essential medium (Eagle), supplemented with 10 % fetal bovine serum. MCF-10A cells were cultured in DMEM/F-12 (Hyclone, USA) supplemented with 10 % horse serum (Cambrex, USA), 10 μg/mL insulin, 2 μg/mL hydrocortisone, 0.01 μg/mL cholera toxin (Sigma-Aldrich, USA) and 0.02 μg/mL epidermal growth factor (EGF) (Sigma-Aldrich, USA).

### Isolated MUC1^−^ESA^+^ subpopulation from MCF-10A cell line

When MCF-10A cell confluence reached about 80 %, single cells were obtained by 0.25 % typsin/EDTA (Gibco, USA) digestion, stained by MUC1-PE (BD Pharmingen, USA) and ESA-FITC (BD Pharmingen, USA). MUC1^−^ESA^+^ subpopulation was sorted by fluorescence-activated cell sorting (FACS, MoFlo, Dako-Cytomation, USA). The rest proportion of MCF-10A cells excluding MUC1^−^ESA^+^ was also sorted as control counterparts. Through FACS sorting, MUC1^−^ESA^+^ subpopulation was highly purified (purity greater than 98 %).

### 2-Dimensional (2-D) clonogenic assay

Cells (200 cells per well) were seeded into a 24-well plate with EpiCult-B serum-free (Stem Cell, USA) and 5 % FBS and 2 × 10^4^ irradiated NIH-3T3 cells. After incubation at 37 °C in 5 % CO_2_ for 8–10 days, colonies of over 50 μm in diameter were counted [27]. The colonies were fixed with acetone: methanol (1:1), stained with Giemsa (Sigma-Aldrich, USA), and observed and photographed under an inverted microscope. Then clonogenic potential was calculated. Clonogenic potential (%) = colony numbers/seeding cell numbers × 100 %. The MCF-10A cells excluding MUC1^−^ESA^+^ were also plated as control.

### 3-Dimensional (3-D) tumor spheroid assay

Cells (100 cells per well) were suspended in EpiCult-B serum-free medium and plated on top of solidified matrigel in a 96-well plate. After 10–14 days’ culture at 37 °C in 5 % CO_2_, colonies over 50 μm in diameter were counted [27]. The test was repeated four times. For immunofluorescence, matrigel mammospheres were embedded in paraffin and cut into slices of 7 μm in thickness for immuno-staining. Immuno-staining of K14-DyLight594 (Biolegend, USA) (1:100), K8-DyLight488 (Biolegend, USA) (1:100) and Dapi (1:1000) was carried out according to their instructions.

### Microarray fabrication and miRNA hybridization

Both human miRNA microarray fabrication and hybridization were performed as described previously [26, 28]. Briefly, miRNA microarray from CapitalBio Corporation (Beijing, China) included 517 mature miRNA sequences [29]. Total RNA was extracted from MUC1^−^ESA^+^ subpopulation with trizol and amplified with NCode™ miRNA amplification system (Invitrogen, USA). After fluorescent labeling of miRNAs, hybridization was conducted at 42 °C overnight. Then data normalization by a normalization factor and clustering was performed based on mean intensity for inter-array comparison. For each sample, two hybridizations were carried out, and each miRNA probe had triplicate dots on the microarray. Significance analysis of microarrays was performed using a two class-unpaired comparison in the SAM procedure Version 2.1 (CapitalBio, China). All the microarray data have been uploaded and submitted to a public repository Gene Expression Omnibus (GEO) database (http://www.ncbi.nlm.nih.gov/geo/query/acc.cgi?acc=GSE68271).

### Real time RT-PCR (qRT-PCR) assay

Total RNA was extracted from sorted cells using RNeasy Micro plus kit (Qiagen, USA), and reverse transcribed into cDNA with standard techniques (ABI, USA)*.* qRT-PCR assay was performed using SYBR® Green PCR Master Mix (ABI, USA). We followed Chen’s protocol for primer design and qRT-PCR [30]. U6 small nuclear RNA (U6 snRNA) was used as an internal control. Its sense and antisense primers were 5′-ctcgcttcggcagcaca-3′ and 5′-aacgcttcacgaatttgcgt-3′. The chosen miRNAs included miR-200c, miR-296, miR-21, miR-373* and miR-122a. The universal sense primer of miRNAs is 5′-gtgcagggtccgaggt-3′. Reverse transcription primer and antisense primer for qRT-PCR are as follows: miR-200c: 5′-gtcgtatccagtgcagggtccgaggtattcgcactggatacgacccatca-3′ and 5′-cgctaatactgccgggtaatg-3′, miR-296: 5′-gtcgtatccagtgcagggtccgaggtattcgcactggatacgacacagga-3′ and 5′-gggccccccctcaatc-3′, miR-21: 5′-gtcgtatccagtgcagggtccgaggtattcgcactggatacgactcaaca-3′ and 5′-gccgctagcttatcagactgatgt-3′, miR-373*: 5′-gtcgtatccagtgcagggtccgaggtattcgcactggatacgacggaaag-3′ and 5′-actcaaaatgggggcgct-3′, miR-122a: 5′-gtcgtatccagtgcagggtccgaggtattcgcactggatacgacacaaac-3′ and 5′-agctggagtgtgacaatggtg-3′. All the qRT-PCR reactions were repeated no less than 3 times.

### miRNA agomir transfection into BCSCs or MaSCs

MCF-7 cells were harvested and digested into single cell suspensions. Obtained cell suspensions were stained with the antibodies (CD24-PE, ESA-FITC and CD44-APC), and ESA^+^CD44^+^CD24^-/low^ BCSCs were sorted with FACS as previously described [26]. Sorted BCSCs (purity greater than 98 %) were suspended in EpiCult-B serum-free medium, and lipofectamine 2000 (Invitrogen, USA) was added together with miR-200c agomir, antagomir (Dharmacon, USA) or miR-control for incubating 24 h. The final concentration of miR-200c agomir, antagomir or miR-control was 30 nM. And miR-200c agomir or antagomir transfection into MaSCs was done in the same manner. The tests were repeated five times.

### Clonogenic ability in vivo

Transfected BCSCs were suspended in EpiCult-B serum-free medium with 25 % matrigel and injected subcutaneously in the mammary fat pads in syngeneic mouse (NSG female, aged 5–6 weeks). The test group was BCSCs transfected with miR-200c agomir with the cell number of 0.5 K, 1 K, 5 K, 10 K, 25 K, 50 K and 100 K. The control group was BCSCs transfected with miR-control with the cell number of 0.5 K, 1 K, 5 K and 10 K. We also set parental BCSCs as a control. Three mice were used for each gradient of cell inoculation. Next, the mice were observed weekly for up to 2 months for tumorigenesis and then sacrificed by cervical dislocation. TIC frequency was calculated and compared using extreme limiting dilution analysis (ELDA, http://bioinf.wehi.edu.au) [31]. All animal procedures were carried out with the approval of the Animal Ethics Committee of the Third Military Medical University.

### Bioinformatics and target prediction

Chromosome localization, sequence analysis and target prediction of the miRNAs were carried out by online programs, picTar (http://pictar.mdc-berlin.de/), miRanda (http://microrna.sanger.ac.uk), targetscan (http://www.targetscan.org), and so on. The mRNAs predicted by three algorithms at least were selected as putative targets. Then mFold Software was used to analyze binding free energy (△G) of hybridization between miRNAs and 3′-UTR complementary sites of mRNAs. Those mRNAs combined with miRNAs with lower free energy at both 5′-70 bp and 3′-70 bp than their average random free energy were deemed accessible to specific miRNAs [32, 33].

### Dual luciferase reporter assay

Through searching for NCBI GenBank database, 3′-UTR sequences of target gene with 100–120 nt in length containing the seed sequence were synthesized. The dangling ends of synthesized fragments were added with XbaI restriction sites (Takara, China). We followed the protocol of our previous work for vector reconstruction and experimental design [34]. Briefly, dual luciferase reporter vectors pGL3-pro and control plasmid pRL-TK (Promega, USA) were used for the assay. Three different 3′-UTR sequences of target gene were synthesized, 3′-UTR 5′ → 3′, 3′-UTR 3′ → 5′ and 3′-UTR 5′ → 3′ without seed sequence. We cloned them into pGL3-pro vector, respectively. The experiment was designed as four groups: test group (pGL3-pro-UTR 5′ → 3′), Con-1 group (pGL3-pro-UTR 3′ → 5′), Con-2 group (pGL3-pro-UTR 5′ → 3′ del), and Con-3 group (empty vector pGL3-pro). We used lipofectamine 2000 to transfect HeLa cells when cell confluence reached 70-80 % in a 24-well plate. Each well of cotransfection reaction contained 200 ng of recombinant pGL3 plasmid, 200 ng of pRL-TK plasmid and 2.5 μL of miR-200c agomir or antagomir. The final concentration of miR-200c agomir or antagomir was 30 nM, 500 μL of liquid in each well. Cells were collected after 48 h of incubation and analyzed for luciferase activity using a dual luciferase reporter system Promega GloMax™ 20/20 (Promega, USA). The luciferase ratio of Firefly/Renilla represented target gene expression. Data of each group are presented as mean ± SD (standard deviation).

### Western blot

Cytoplasmic protein was extracted according to the manufacturer’s instructions (Sigma-Aldrich, USA). Western blot was performed as described in detail in the earlier report [35]. Primary antibodies used in this study included anti-human programmed cell death 10 (PDCD10) (Abcam, USA).

### Lentivirus infection

Lentiviruses were produced and purified as described previously [36]. Reconstructed PDCD10 plasmid was generated by PCR-cloning full-length human PDCD10 cDNA (AF022385) into the EcoRI/BamHI sites of lentivirus vector pCDH (System biosciences, USA). Primers for PCR are 5′-ccggaattcatgaggatgacaatggaa-3′ and 5′-cgcggatcccaggccacagttttgaag-3′. Recombinant plasmid was cotransfected with packaging plasmids into HEK-293 T cells to produce lentivirus particles (Lenti-PDCD10). Active viruses were mixed with sorted BCSCs supplemented with 8 μg/mL Polybrene (Sigma, USA), and seeded to a 3D matrigel culture system with EpiCult-B serum-free medium. As for the rescue experiment, Lenti-PDCD10, Polybrene and miR-agomir were mixed in EpiCult-B serum-free medium beforehand, and then added into the matrigel culture system. The final concentration of miR-agomir or miR-control was 30 nM.

## Results

### Stemness assessment of MUC1^−^ESA^+^ cells

**Fig. 1:**
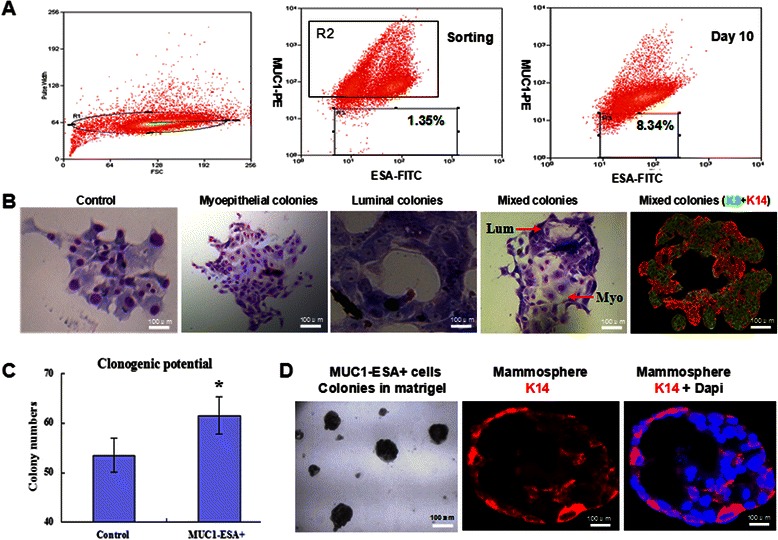
Tumor-initiation ability of MUC1^−^ESA^+^ cells sorted from mammary epithelium. **a**. MUC1^−^ESA^+^ subpopulation accounts for 1.35 % of MCF-10A cells when being sorted, and 8.34 % in serum-free culture on day 10. **b**. In the 2-D culture, sorted MUC1^−^ESA^+^ cells show three types of colonies including myoepithelial, luminal and mixed colonies, while the control cells (MCF-10A excluding MUC1^−^ESA^+^) displayed a unique type of colonies. **c**. The number of colonies and histogram of panel B (*, compared with the control, *n* = 6, *P* < 0.05). **d**. In the 3-D matrigel culture, sorted MUC1^−^ESA^+^ cells proliferate into colonies with duct-like structures and myoepithelial marker K14-DyLight 594 expression

FACS analysis showed that MUC1^−^ESA^+^ subpopulation in MCF-10A cells accounted for 1–1.5 % (Fig. 1a). For 2-D clonogenic assay after 10 days of culture, sorted MUC1^−^ESA^+^ cells showed multiple morphological colonies, at least three types of mammary epithelial cell colonies including pure luminal colonies, pure myoepithelial colonies and mixed colonies. Immuno-staining confirmed that mixed colonies had multi-component including myoepithelial cells (K14-DyLight594) and luminal cells (K8-DyLight488). The rest MCF-10A cells excluding MUC1^−^ESA^+^ (non-stem MCF-10A) showed a unique type of colonies (Fig. 1b).

The clonogenic potential of MUC1^−^ESA^+^ cells was 61.50 ± 3.77 %. However, the clonogenic potential of non-stem MCF-10A cells was 53.50 ± 3.44 %. MUC1^−^ESA^+^ cells had significantly higher clonogenic potential (*P* < 0.05, Fig. 1c).

After 10 days of 3-D matrigel culture, MUC1^−^ESA^+^ cells also grew into mammospheres. After immuno-staining with myoepithelial marker K14-DyLight594 (1:100), mammospheres showed ductal-like structure (Fig. 1d). However, mammospheres of non-stem MCF-10A cells did not show duct-like structures (Additional file 1: Figure S1a). Additionally, mammospheres on day 10 were digested and analyzed by FACS, showing that the proportion of MUC1^−^ESA^+^ cells increased to 8.34 % (Fig. 1a). These results indicated that MUC1^−^ESA^+^ cells possessed stemness properties of multi-potential differentiation and clonogenesis. MaSCs could be obtained by sorting MUC1^−^ESA^+^ subpopulation from MCF-10A cell line.

### miRNA profile of BCSCs distinct from MaSCs

**Fig. 2:**
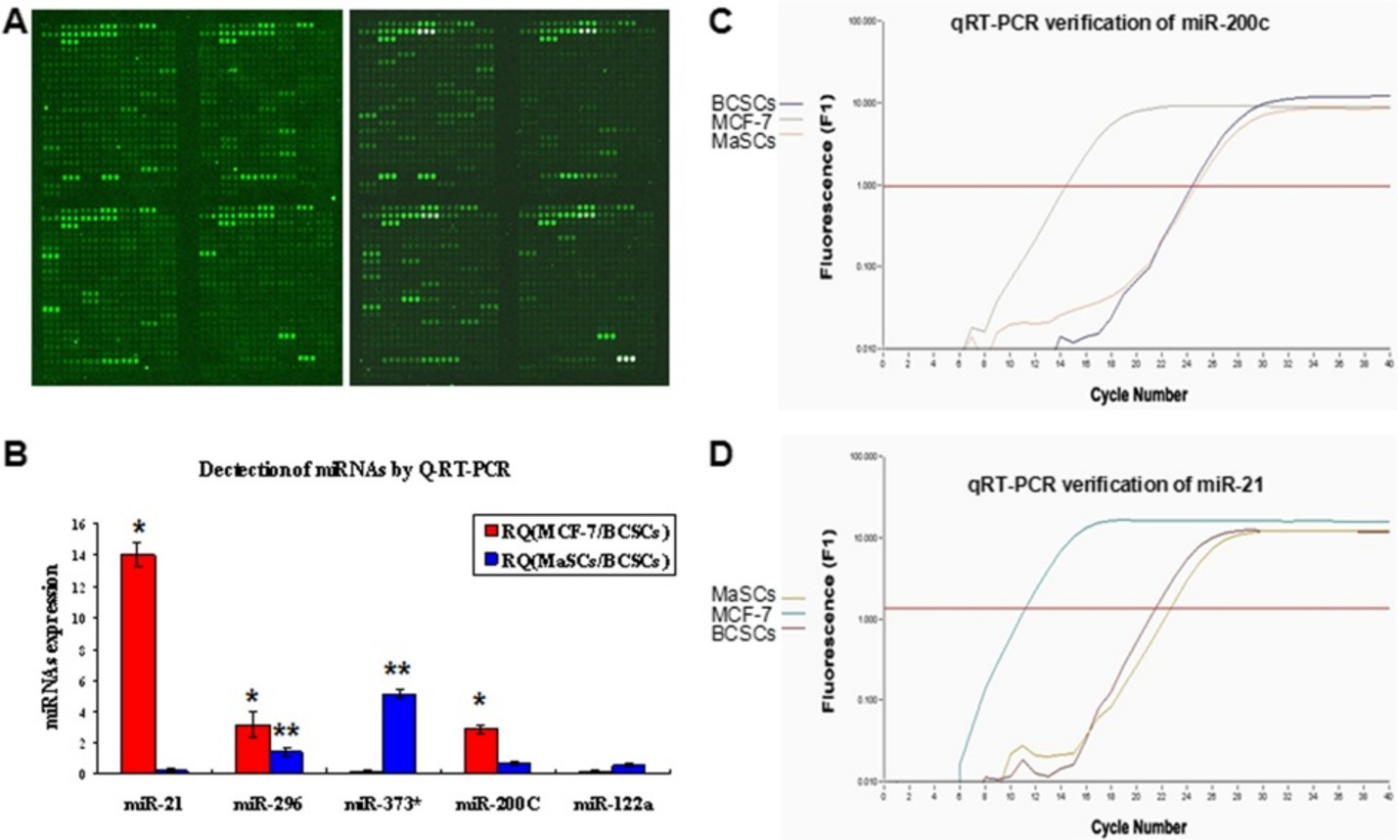
Microarray analysis of miRNA expression in MaSCs and qRT-PCR verification. **a**. Duplicate microarray analyses of miRNA expression in MaSCs. Triplicate dots on the microarray exhibit consistent signal intensity. **b**. Relative quantitative (RQ) expressions of 5 miRNAs in MCF-7/BCSCs/MaSCs shown as mean ± standard deviation (SD). (*, The expressions of miR-21, miR-296 and miR-200c are significantly higher in MCF-7 than in BCSCs. **, the expression of miR-373* is significantly higher in MaSCs than in BCSCs). **c**. Amplification curve of miR-200c in MCF-7/BCSCs/MaSCs. Comparison of miR-200c expressions tends to be MaSCs < BCSCs < MCF-7. **d**. Amplification curve of miR-21 in MCF-7/BCSCs/MaSCs. Comparison of miR-21 expressions tends to be MaSCs < BCSCs < MCF-7

The internal control U6 snRNA dots on all microarrays exhibited consistent signal intensity and the signal of all the detected dots in the replicate microarrays showed a high correlation efficiency (*R* = 0.9616 ± 0.0244), indicating the repetitiveness and reproducibility of the microarrays. Collectively, a profile of 72 mature miRNAs was detected (with signal value above 800) in MaSCs (Fig. 2a).

In our previous study, we obtained miRNA profile in BCSCs from breast cancer cell line MCF-7 [26]. Since it is hypothesized that BCSCs initiate from MaSCs, BCSC-related miRNAs distinct from MaSCs could clarify the mechanism of mammary tumorigenesis. We compared miRNome data between BCSCs and MaSCs, and obtained 12 differential miRNAs with fold change more than 3 times. Among them, 8 miRNAs were upregulated in BCSCs (miR-25, let-7f, miR-342, miR-103, miR-21, miR-16, miR-200c and miR-122a) and 4 miRNAs were downregulated in BCSCs (miR-345, miR-155, miR-205 and miR-494) (Table 1).

**Table 1:** Distinct miRNAs between BCSCs and MaSCs in microarray

Upregulated miRNA in BCSCs	Fold change	Downregulated miRNA in BCSCs	Fold change
miR-122a	4.731	miR-494	0.099
miR-200c	4.022	miR-205	0.100
miR-16	3.703	miR-155	0.319
miR-21	3.675	miR-345	0.323
miR-103	3.647		
miR-342	3.615		
let-7f	3.365		
miR-25	3.267		

### Verification of miRNA microarray by qRT-PCR assay

To verify the results of miRNA microarray, miRNA qRT-PCR assay was performed. Besides the miR-200c, miR-21 and miR-122a upregulated in BCSCs compared with MaSCs, we also chose miR-296 and miR-373* which were respectively downregulated and upregulated in BCSCs compared with paternal MCF-7 cells in our prior study [26], and highly associated with the development of human embryonic stem cells [37, 38]. Thus, a total of 5 miRNAs (miR-200c, miR-296, miR-21, miR-373* and miR-122a) were selected to qRT-PCR assay among three types of cells, BCSCs, MaSCs and MCF-7. The relative quantitative (RQ) miRNA expression of MCF-7/BCSCs and MaSCs/BCSCs were shown as mean ± SD (Table 2, Fig. 2b). Amplification curve of representative miRNAs were also displayed (examples in Fig. 2c and d). As a result, there were 10 qRT-PCR reactions of 5 miRNAs between BCSCs, MaSCs and MCF-7 cells. Among them, 8 reactions were consistent with miRNA microarray except for miR-296 and miR-200c in MCF-7/BCSCs (Table 2). Collectively, 80 % consistency in these data indicated that the results of miRNA microarray in our study were highly reliable. According to our hypothesis that MaSCs is the source of BCSCs which proliferate and differentiate into breast cancer cells, miRNAs with consecutive changes from MaSCs to BCSCs to MCF-7 cells would more likely be the essential regulators of self-renewal of BCSCs. Thus, miR-373*, miR-21 and miR-200c could be the candidates. Here, we chose miR-200c for following research since it has been proven to be a critical regulator of BCSCs in primary tumor tissues [10].

**Table 2:** Relative expression of miRNAs between MCF-7/BCSCs/MaSCs

miRNAs	△CT	RQ	Chip	△CT	RQ	Chip
	BCSCs-MCF7	MCF7/BCSCs	MCF7/BCSCs	MaSCs-BCSCs	MaSCs/BCSCs	MaSCs/BCSCs
U6 RNA	3.303 ± 0.297	8.154 ± 0.516		−0.937 ± 0.182	0.553 ± 0.064	
miR-21	7.390 ± 0.089	14.052 ± 0.753	4.75	3.127 ± 0.809	0.259 ± 0.096	0.272
miR-296	4.990 ± 0.255	3.133 ± 0.830	0.191	−0.427 ± 0.197	1.403 ± 0.310	1.025
miR-373*	0.240 ± 0.615	0.150 ± 0.052	0.162	1.607 ± 0.134	5.092 ± 0.310	1.696
miR-200c	4.987 ± 0.290	2.913 ± 0.278	0.581	−1.497 ± 0.298	0.705 ± 0.106	0.249
miR-122a	0.280 ± 0.759	0.158 ± 0.064	0.020	−1.803 ± 0.412	0.585 ± 0.091	0.211

### Role of miR-200c in regulating stemness of BCSCs and MaSCs

**Fig. 3:**
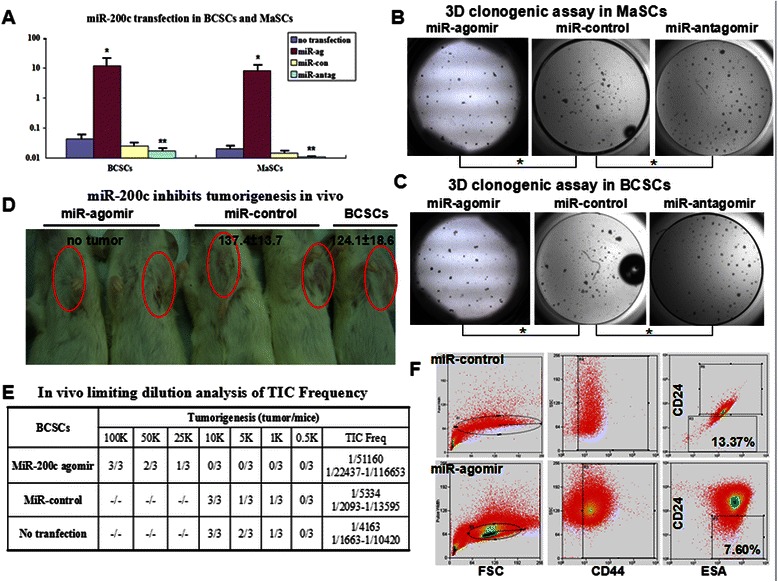
miR-200c inhibits the self-renewal of BCSCs and MaSCs. **a**. In qRT-PCR assay, miR-200c agomir significantly upregulates miR-200c expression in both BCSCs and MaSCs (*, *P* < 0.01); miR-200c antagomir significantly downregulates miR-200c expression in both BCSCs and MaSCs (**, *P* < 0.01). **b**. In MaSCs, miR-200c agomir significantly decreases the colonies (*P* < 0.01, *n* = 5) while miR-200c antagomir significantly increases the colonies (*P* < 0.01, *n* = 5). **c**. In BCSCs, miR-200c agomir significantly decreases colonies (*P* < 0.01, *n* = 5) while miR-200c antagomir significantly increases colonies (*P* < 0.01, *n* = 5). **d**. No tumor was observed in the test group (miR-200c agomir) in 2 months after inoculation of 10 K cells. In the miR-control group and parental BCSC group, average tumor volumes are 137.4 ± 13.7 mm^3^ and 124.1 ± 18.6 mm^3^, respectively. **e**. A limiting dilution assay for tumorigenesis in vivo and TIC Frequency calculation. **f**. Surface markers (ESA^+^CD44^+^CD24^-/low^) of BCSCs were detected on day 10 after transfecting miR-agomir or miR-control

To test biological function of miR-200c, we introduced miR-200c agomir and miR-200c antagomir into both BCSCs and MaSCs, respectively. In qRT-PCR assay, miR-200c was expressed much higher after miR-200c agomir transfected into BCSCs and MaSCs than the control (*P* < 0.01) analyzed by Tamhane’s test or non-parametric statistics analysis (SPSS 18.0). miR-200c expression reached 281 and 408 times higher in BCSCs and MaSCs, respectively. And miR-200c antagomir significantly downregulated miR-200c expression in BCSCs and MaSCs than the control (*P* < 0.01). The fold changes were 0.40 and 0.52, respectively (Fig. 3a).

To test whether miR-200c agomir is related to self-renewal of MaSCs and BCSCs, we utilized the matrigel 3-D culture system. From seeding cells of the same number, MaSCs transfected with miR-200c agomir resulted in significantly fewer mammospheres (28.8 ± 2.05) (*P* < 0.01, *n* = 5), and miR-200c antagomir significantly increased mammospheres (61.80 ± 5.54) (*P* < 0.01, *n* = 5) than those transfected with miR-control (42.20 ± 3.35) (Fig. 3b). The similar result was found in BCSC experiment. BCSCs transfected with miR-200c agomir resulted in significantly fewer mammospheres (22.2 ± 1.92) than those transfected with miR-control (40.60 ± 3.05) (*P* < 0.01, *n* = 5). In contrast, miR-200c antagomir significantly increased mammospheres (60.80 ± 5.40) (*P* < 0.01, *n* = 5, Fig. 3c). These results suggest that miR-200c could inhibit the self-renewal of both MaSCs and BCSCs.

Next, to directly test whether overexpression of miR-200c could affect tumor initiating ability of BCSCs in vivo, we performed limiting dilution assay for mammary tumors. Basically, BCSCs transfected with miR-200c agomir gave rise to smaller tumors than the control at the same number of inoculation. In gradient assay, tumors could be observed in every group when 10 K BCSCs transfected with miR-control were inoculated subcutaneously, while 10 K BCSCs transfected with miR-200c agomir could not grow into a tumor. The average tumor volumes at two months were 124.1 ± 18.6 mm^3^ and 137.4 ± 13.7 mm^3^ in 10 K parental BCSCs and miR-control transfected BCSCs, respectively (Fig. 3d). To reach a similar tumorigenicity, the quantity of required BCSCs transfected with miR-control was 10 K while that of BCSCs transfected with miR-200c agomir was 100 K. We calculated the TIC frequency of these groups by ELDA in addition to specifying the inoculation cell numbers and tumor growth. As a result, miR-200c agomir led to 9.59 times lower tumor initiating ability compared with miR-control (1/51160 vs. 1/5334) (Fig. 3e). Taken together, miR-200c downregulation is required by BCSCs for survival and expansion both in vitro and in vivo.

Therefore, we wondered if miR-200c had some effects on sustaining the “stemness” of BCSCs. We detected the surface markers (ESA^+^CD44^+^CD24^-/low^) of BCSCs on day 10 after transfecting miR-agomir or miR-control. The proportion of stem cells decreased in the miR-200c transfection group compared with that in the miR-control transfection group (7.60 vs 13.37 %, Fig. 3f).

### Verification of PDCD10 as miR-200c target

**Fig. 4:**
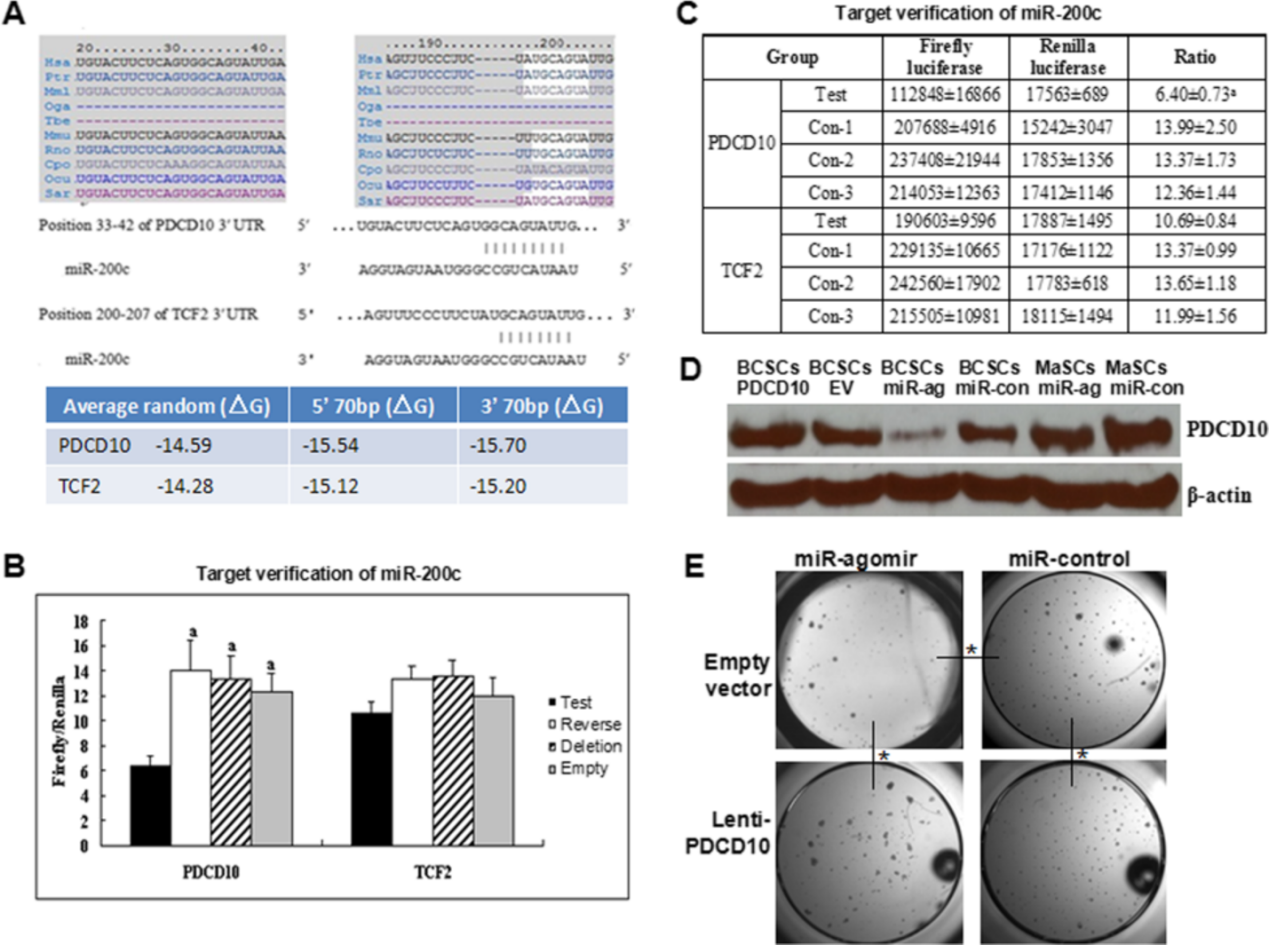
Verification of PDCD10 as a target of miR-200c. **a**. The binding sites of PDCD10 and TCF2-to miR-200c, and mFOLD analysis of free energy. **b**. Histogram of dual luciferase assay (a, compared with test group, *P* < 0.05). **c**. Experimental data of dual luciferase assay (a, compared with control groups, *P* < 0.05). **d**. In western blot assay, compared with empty vector (EV), Lenti-PDCD10 dramatically increases PDCD10 expression in BCSCs, and compared with miRNA control (miR-con), miR-200c agomir (miR-ag) dramatically decreases PDCD10 expression in BCSCs and MaSCs. **e**. In the 3-D matrigel culture, miR-200c agomir decreases, and Lenti-PDCD10 increases mammospheres of BCSCs compared with miR-control (*P* < 0.01, *n* = 5). Lenti-PDCD10 rescues mammospheres inhibited by miR-200c agomir (*P* < 0.01, *n* = 5)

We listed 24 potential targets of miR-200c, including TMEFF2, TIEG, TGFB1I4, TDE2, TCF8, TCF2, TBP, SYVN1, PDCD10, SFRS2, SFRS1, PTPN13, RAP2C, RAP1B, RAB7, RAB2, GATA4, FGFR2, ESRRG, EPS8, EIF5B, EIF3S1, EIF2B5 and APRIN. These potential targets involved in oncogenes, anti-oncogenes, transcription factors and DNA repair, cell cycle regulation, miRNA processing and signal transduction. Then mFOLD analysis showed that two of them, PDCD10 and TCF2, could be the putative targets of miR-200c. The binding free energies between PDCD10 and miR-200c at both 5′-70 bp and 3′- 70 bp were −15.54 and −15.70, respectively, lower than average random free energy of PDCD10 (−14.59). And the binding free energies between TCF2 and miR-200c at both 5′ 70 bp and 3′ 70 bp were −15.12 and −15.20, respectively, lower than average random free energy of TCF2 (−14.28). Also, miR-200c showed broadly conserved binding sites with PDCD10 and TCF2 in different species (Fig. 4a). Thus, PDCD10 and TCF2 were chosen for further study.

For miR-200c target PDCD10 verification, the ratios of firefly/renilla in dual luciferase assaywere similar between reverse, deletion and empty vector control groups (13.99 ± 2.50, 13.37 ± 1.73 and 12.36 ± 1.44), whereas the relative luciferase activity (6.40 ± 0.73) was significantly lower in the test group (*P* < 0.05) (Fig. 4b and c). For miR-200c target TCF2 verification, no significant difference was found between the test group and the three control groups (Fig. 4b and c).

The protein change of PDCD10 during miR-200c agomir transfection had been tested by western blot. Compared with the control group (miR-control), PDCD10 proteins were dramatically reduced in BCSCs and MaSCs, respectively (Fig. 4d).

To sufficiently prove that PDCD10 is the target of miR-200c, we performed more experiments. First, since miR-200c agomir inhibited mammospheres of BCSCs and MaSCs, and miR-200c antagomir demonstrated opposite effects, overexpresion of PDCD10 should promote the “stemness” of BCSCs. Actually, the experiment of upregulated PDCD10 by lentivirus vector (Fig. 4d) showed more mamaospheres in BCSCs (60.40 ± 5.03) as expected compared with empty vector transfection (42.20 ± 3.49) (*P* < 0.01, *n* = 5, Fig. 4e). Second, rescue experiment was delivered in tumor spheroid assay of miR-200c agomir in BCSCs. BCSCs cotransfected with Lenti-PDCD10 and miR-200c agomir showed more mammospheres (52.60 ± 4.67) compared with BCSCs transfected with miR-200c agomir alone (26.40 ± 2.07) (*P* < 0.01, *n* = 5, Fig. 4e). From these results, we believe that PDCD10 is responsible for miR-200c-mediated decrease in mammospheres in BCSCs.

## Discussion

As molecules that regulate biological growth and development, miRNAs show aberrant expression in many malignant tumors. MiRNAs also play a vital role in maintaining self-renewal and multi-directional differentiation of human embryonic stem cells and other adult stem cells [20, 39, 40]. These intriguing questions remain to be answered in looking for the source of breast cancer. Although some reports showed that differentiated breast cancer cells could be reprogrammed into BCSCs [41], it is hypothesized that BCSCs could initiate from accumulating mutations of MaSCs [11–13]. For example, there are considerable similarities between basal-like and BRCA1-mutated breast cancers, and these cancers arise from transformation of a basal cell in normal breast epithelium through BRCA1 dysfunction [42]. Therefore, screening of miRNA profiles in BCSCs and MaSCs seems significant to elucidate the role and mechanism of miRNAs in tumorigenesis of breast cancer.

MUC1^−^/ESA^+^ cells have been demonstrated to function as stem cells of terminal duct lobular units in the human breast [15]. We successfully sorted MUC1^−^ESA^+^ cells from MCF-10A cells. The subsequent experiments showed that MUC1^−^ESA^+^ cells had the ability to form both acinar- and duct-like colonies, indicating their stemness and capability of multi-directional differentiation. To our best knowledge, it is the first report to isolate MaSCs from a human mammary cell line.

Growing evidence shows the involvement of miRNAs in mammary biology and breast cancer. For instance, miR-206 expression was higher in ERalpha-negative MB-MDA-231 cells than in ERalpha-positive MCF-7 cells [43], and enforced expression of miR-125a or miR-125b led to coordinate suppression of ERBB2 and ERBB3 in the human breast cancer cell line SKBR3 [44]. Furthermore, miR-27b could be one of the causes of up-regulation of the drug-metabolizing enzyme CYP1B1 in cancerous tissues [45]. Then, as a tumor suppressor in breast cancer cells, miR-17-5p regulated breast cancer cell proliferation by inhibiting the translation of AIB1 mRNA [46]. Ma et al. [47] found that the expression of miR-10b was significantly increased in metastatic breast cancer cell line, indicating the possible role of miR-10b in facilitating metastasis. The down-regulated expression of miR-125b in breast cancer cells suggests the possible anti-tumor effect of miR-125b [40]. As for miR-200c, several reports showed that miR-200c played important roles in all kinds of biological features in breast cancer cells. For instance, miR-200c upregulation in MCF-7 led to reduced expression of transcription factor 8 and increased expression of E-cadherin [48]. The low- or non-expression of miR-200c may lead to the invasion and migration of breast cancer cells [49, 50]. Dykxhoorn et al. [51] found in a mouse model of breast cancer that miR-200 family could suppress the expression of Zeb2, a transcription inhibiting gene, and enhance the expression of E-cadherin, thus inhibiting endothelial mesenchymal transformation (EMT). In treatment of breast cancer cells, miR-200c was also reported to sensitize apoptosis [52], chemotherapy [53, 54], radiotherapy [55, 56] and trastuzumab targeted therapy [57].

Recently, a few studies have reported miRNA expression in BCSCs. Shimono [10] found that 37 miRNAs were upregulated or downregulated in BCSCs compared with non-tumorigenic breast cancer cells. In this report, miR-200c played important regulatory roles in maintaining the function of BCSCs by downregulating BMI 1 and in inhibiting EMT. Another research showed that let-7 downregulation in human BCSCs affected the self-renewal ability of stem cells via regulating its target gene Ras, and changed the differentiation ability of stem cells by regulating HMGA2 expression [21]. However, there are few reports on the different miRNA profiles between MaSCs and BCSCs.

In this study, we successfully obtained miRNome data of BCSCs and MaSCs, and found 12 differentially expressed miRNAs. Thereafter, miR-200c was chosen and analyzed for its function in regulating self-renewal of MaSCs and BCSCs. By high-throughput miRNA microarray and qRT-PCR assay, miR-200c in BCSCs was confirmed to be downregulated 2.913 times compared with that in MCF-7 and upregulated 1.418 times compared with that in MaSCs (Table 2). Consecutive upregulation of miR-200c from MaSCs to BCSCs to MCF-7 implied the essential role of miR-200c in the initiation of mammary tumor. Functional assays indicated the critical role of miR-200c in suppressing the self-renewal of human BCSCs and MaSCs. In BCSC study, we not only conducted the clonogenic assay in vitro, but also calculated TIC frequency in vivo. By detecting the surface markers (ESA^+^CD44^+^CD24^-/low^) in vitro, we found that miR-200c decreased the proportion of stem cells. However, as the agomir of miR-200c could not be inserted into genomic DNA like lentivirus vector, its function was time-dependent in vivo. Theoretically, the transfected BCSCs would differentiate into tumor cells in formed tumors several weeks later and return to the same proportion as was in the control tumors [58]. We could not determine whether miR-200c had an effect on the prevalence of the stem cell population in these tumors. Nevertheless, both in vitro clonogenic assay and in vivo tumorigenesis assay showed that miR-200c functioned as an anti-oncogene. Collectively, the increase of miR-200c in MCF-7 could result from the stemness loss of BCSCs and their uncontrollable proliferation and differentiation.

In the present research, miR-200c was found downregulated 1.808 times in MaSCs compared with parental MCF-10A cells (GSE68271, Additional file 1: Figure S1b). Since ectopic expression of miR-200c suppressed the tumorigenic ability of BCSCs, the downregulation of miR-200c in MaSCs suggests that MaSCs also possess a tendency of tumorigenesis compared with MCF-10A cells. These results are consistent with the findings of other groups [6, 59, 60]. Interestingly, we found that miR-200c level was modestly lower in MaSCs than in BCSCs (Fold Change 0.705). It is appreciated that the tumorigenic ability of CSCs is stronger than that of normal stem cells, and high activity of self-renewal does not completely correlate with tumorigenesis [6, 59, 60]. Our results indicate that miR-200c plays a critical role in the self-renewal of MaSCs and BCSCs. To our best knowledge, this is the first time to find that miR-200c could be involved in malignant transformation of MaSCs into BCSCs at the level of cell lines. Our findings here support previous reports that miR-200c is critical in regulating the self-renewal of BCSCs in primary tumor tissue [10]. Of course, we did not verify these findings in MCF-10A derived tumor cell MCF10DCIS.com, but a future research would be expected.

Furthermore, we searched and verified PDCD10, a new target of miR-200c in the present study. Bioinformatics and prediction programs have become preferential methods to explore the function of miRNAs [61, 62]. The genes possibly regulated by BCSC-related miRNAs should be involved in both tumorigenesis and stem cell maintenance. PDCD10 is an apoptosis-related gene of 1,218 bp in length located on chromosome 3q26.1, highly conserved in different species. As an important apoptosis regulator, PDCD10 is upregulated in various tumors [63–65]. PDCD10 is also involved in angiogenesis and vascular reconstruction and closely associated with the prognosis of cancer patients [66]. In dual-luciferase reporter assay and western blot, we observed that miR-200c inhibited PDCD10 expression in both BCSC and MaSC subpopulations. In an ischemic preconditioning (IPC) model, PDCD10 was confirmed to be significantly reduced in preconditioned mesenchymal stem cells (MSCs) [67]. In the present study, PDCD10 promoted the self-renewal of BCSCs. Since miR-200c inhibited the stemness of BCSCs/MaSCs and PDCD10 simultaneously, PDCD10 could be a possible mechanism mediated by miR-200c in stemness regulation of breast tumorigenesis and malignant transformation.

Recently, reports showed that both small interfering RNAs (siRNAs) and miRNAs could have off-target effects (OTEs) [68, 69]. In the present study, we obtained an overexpression of miR-200c to an extent of 300–400 times. However, there was no evidence of OTEs, since miR-200c antagomir showed opposite effects against miR-200c agomir in functional analysis. Moreover, almost at the same time as our research, an article preliminarily demonstrated that PDCD10 was a target of miR-200C, in which only dual-luciferase method was used [70]. In our research, we also performed verification experiments including western blot, PDCD10 overexpression and rescue experiment to confirm it was a real target of miR-200c. Thus, the present study indicates that PDCD10 is a new mechanism underlying miR-200c stemness maintenance in both MaSCs and BCSCs.

The BCSC-related miRNAs mentioned above could provide novel insights into BCSCs initiation, self-renewal and stemness maintenance. Future work should include verification of the putative targets of other BCSC-related miRNAs identified here.

## Conclusions

In summary, we obtain the miRNome data of BCSCs and MaSCs, and a series of functional researches show that PDCD10 is a target gene of miR-200c and also a possible mechanism by which miR-200c regulates the stemness of BCSCs and MaSCs. Our identification of BCSC-related miRNAs should be a starting point to explore the mechanism of malignant transformation of MaSCs into BCSCs, which may provide new insights into the complex picture of BCSCs and assist cancer biologists and clinical oncologists in designing and testing novel therapeutic strategies.

## Additional file

Additional file 1: Figure S1. Additional materials. a. In the 3-D matrigel culture, MCF-10A cells excluding MUC1^−^ESA^+^proliferate into colonies. Mammospheres of non-stem MCF-10A cells do not show duct-like structures. b. miR-200c is downregulated 1.808 times in MaSCs compared with non-stem MCF-10A cells. (TIFF 1147 kb)

## Abbreviations

CSCs: Cancer stem cells
BCSCs: Breast cancer stem cells
MaSCs: Mammary epithelial stem cells
TICs: Tumor initiating cells
ELDA: Extreme limiting dilution analysis
PDCD10: Programmed cell death 10
