# Supplementary figures and images for: Essential role of miR-200c in regulating self-renewal of breast cancer stem cells and their counterparts of mammary epithelium
Source: BMC Cancer. 2015 Sep 23;15:645. doi: 10.1186/s12885-015-1655-5 (PMC4581477; doi:10.1186/s12885-015-1655-5)

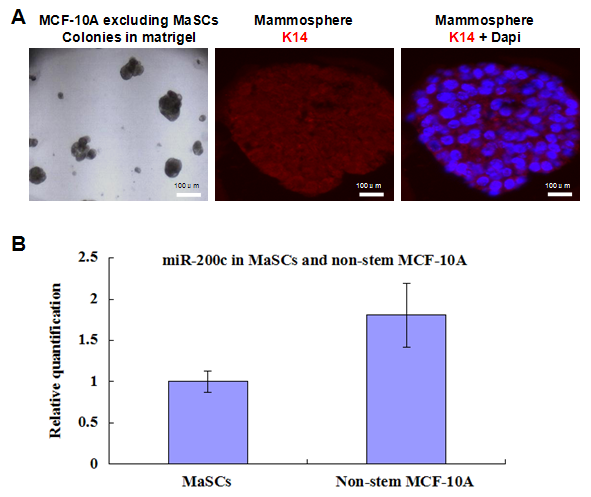

Supplement: Additional file 1: Figure S1. — Additional materials. a. In the 3-D matrigel culture, MCF-10A cells excluding MUC1−ESA+proliferate into colonies. Mammospheres of non-stem MCF-10A cells do not show duct-like structures. b. miR-200c is downregulated 1.808 times in MaSCs compared with non-stem MCF-10A cells. (TIFF 1147 kb) [file 12885_2015_1655_MOESM1_ESM.tiff]
